# Host-specific factors affect the pathogenesis of adverse reaction to metal debris

**DOI:** 10.1186/s12891-019-2578-0

**Published:** 2019-05-04

**Authors:** Lari Lehtovirta, Aleksi Reito, Olli Lainiala, Jyrki Parkkinen, Harry Hothi, Johann Henckel, Alister Hart, Antti Eskelinen

**Affiliations:** 1 Faculty of Medicine and Health Technology, University of Tampere, Tampere, Finland; 2 Coxa Hospital for Joint Replacement, Tampere, Finland; 3 Fimlab Laboratories Oy, Tampere, Finland; 4 University College London, London, UK

**Keywords:** Metal-on-metal, ASR, ARMD, ALVAL, Pseudotumor, Pseudotumour, Patient susceptibility, Host response

## Abstract

**Background:**

Adverse Reaction to Metal Debris (ARMD) is a major reason for revision surgeries in patients with metal-on-metal (MoM) hip replacements. Most failures are related to excessively wearing implant producing harmful metal debris (extrinsic factor). As ARMD may also occur in patients with low-wearing implants, it has been suggested that there are differences in host-specific intrinsic factors contributing to the development of ARMD. However, there are no studies that have directly assessed whether the development of ARMD is actually affected by these intrinsic factors.

**Methods:**

We included all 29 patients (out of 33 patients) with sufficient data who had undergone bilateral revision of ASR MoM hips (58 hips) at our institution. Samples of the inflamed synovia and/or pseudotumour were obtained perioperatively and sent to histopathological analysis. Total wear volumes of the implants were assessed. Patients underwent MARS-MRI imaging of the hips preoperatively. Histological findings, imaging findings and total wear volumes between the hips of each patient were compared.

**Results:**

The difference in wear volume between the hips was clinically and statistically significant (median difference 15.35 mm^3^, range 1 to 39 mm^3^, IQR 6 to 23 mm^3^) (*p* < 0.001). The median ratio of total wear volume between the hips was 2.0 (range 1.09 to 10.0, IQR 1.67 to 3.72). In majority of the histological features and in presence of pseudotumour, there were no differences between the left and right hip of each patient (*p* > 0.05 for all comparisons). These features included macrophage sheet thickness, perivascular lymphocyte cuff thickness, presence of plasma cells, presence of diffuse lymphocytic infiltration and presence of germinal centers*.*

**Conclusions:**

Despite the significantly differing amounts of wear (extrinsic factor) seen between the sides, majority of the histological findings were similar in both hips and the presence of pseudotumour was symmetrical in most hips. As a direct consequence, it follows that there must be intrinsic factors which contribute to the symmetry of the findings, ie. the pathogenesis of ARMD, on individual level. This has been hypothesized in the literature but no studies have been conducted to confirm the hypothesis. Further, as the threshold of metal debris needed to develop ARMD appears to be largely variable based on the previous literature, it is likely that there are between-patient differences in these intrinsic factors, ie. the host response to metal debris is individual.

**Electronic supplementary material:**

The online version of this article (10.1186/s12891-019-2578-0) contains supplementary material, which is available to authorized users.

## Background

Adverse Reaction to Metal Debris (ARMD) continues to be a major reason for revision surgeries in patients with Metal-on-Metal (MoM) hip replacements [1, 2]. ARMD consists of very variable and heterogenous findings and symptoms. Patients may experience strong pain and discomfort or be completely asymptomatic [3]. Radiologically, fluid-filled cystic lesions or solid inflammatory soft-tissue masses termed pseudotumors can be found on some patients, both symptomatic and asymptomatic [4, 5]. Microscopical findings in periprosthetic tissue range from mild macrophage infiltration to deep soft-tissue necrosis with heavy lymphocyte infiltration [6–8]. In summary, there is a high between-subject variability with regard to symptoms, clinical findings and histological presentation of the tissues in patients with ARMD.

Factors that affect the development of ARMD can be divided into extrinsic and intrinsic. The amount of wear debris and physicochemical properties of the particles are examples of extrinsic factors. Intrinsic factors, such as individual differences in innate and adaptive immune responses to metal wear debris, can be collectively referred to as host response [9]. Several retrieval studies have investigated extrinsic factors, most importantly implant wear, and their association to ARMD. Many studies have shown that implant wear is a risk factor for the development of ARMD [10–12]. However, adverse reactions have also been observed in patients with low wearing hip implants in several studies [8, 13–15]. In their systematic review, Campbell et al. concluded that no clear dose-response relationship between wear and ARMD could be established due to the heterogeneity of the findings in the included studies [16]. Studies that have investigated association between the histopathological features of ARMD and wear or indirect markers of wear, such as synovial fluid or whole blood metal ion concentrations, have also yielded inconsistent results [6, 8, 14, 17–23]. The lack of a clear association between extrinsic factors and the development of ARMD could be due to a remarkable role of intrinsic factors affecting the pathogenesis. In fact, the contribution of host-specific factors and presence of patient susceptibility has been suggested in numerous previous studies based on the between-subject discrepancy in the amount of wear debris needed to result in ARMD and implant failure [8, 13, 14, 24–28]. Further, it has been suggested that women are more susceptible than men, possibly due to previous exposure to metals from jewelry [14, 15, 29]. However, to the best of our knowledge, there are no studies that would have actually investigated whether intrinsic factors affect the pathogenesis of ARMD in patients with MoM hips.

In the present study, we aimed to indirectly investigate whether host-specific intrinsic factors affecting the pathogenesis of ARMD exist in a cohort of patients with bilateral ASR hips, both of which were revised for ARMD. Host response was investigated by comparing both histological findings and the amount of bearing surface wear volume (extrinsic factor) between each patient’s left and right hips. Each hip served as a control for the other. If the tissue response between the hips was similar (low within-subject variability) despite differing amount of wear debris between the sides (difference in an extrinsic factor), it would indicate the presence of intrinsic factors contributing to the similarity of the tissue response (Additional file 1). We had three hypotheses: 1) there is significant congruence in histological findings between the hips of each patient (low within-subject variability) despite differing amount of wear between the hips, indicating the contribution of intrinsic factors in the pathogenesis, 2) histological findings characteristic of the innate immune response or direct cytotoxic effects of metal debris (macrophages, granulomas and necrosis) would differ between the sides in response to wear debris and 3) components of the individual adaptive immune response (lymphocytes, germinal centers and plasma cells) would be congruent between the sides as a result of contribution of intrinsic factors.

## Methods

### Study design

One thousand thirty-six Articular Surface Replacement (ASR) MoM hip replacements (Depuy Orthopaedics, Warsaw, IN, USA) were performed in 887 patients at our institution between March 2004 and December 2009. By the end of September 2016, 316 patients had been revised. Of these, 33 patients have undergone bilateral revision. Four of these patients were excluded due to missing tissue samples thus leading to 29 patients being included in our study (58 hips). Flow chart of the patient selection is available as a supplement (Additional file 2). All patients had the same head-cup-combination on both sides: five patients had bilateral ASR hip resurfacing and 24 patients had ASR XL stemmed total hip replacements bilaterally. Simultaneous bilateral hip revision was performed for two patients, and the remaining 27 patients’ bilateral revision surgeries were performed sequentially. Revision operations have been described in detail in our previous publication [30]. Patient demographics and indications for revision surgery are presented in Table 1. Surgery was performed by or under the direct supervision of 10 senior orthopedic surgeons. All patients gave written informed consent to participate in this study that was approved by the ethical committee of Pirkanmaa Hospital District (R11006).

**Table 1:** Reasons for revision surgery

Reasons for revision surgery	
Progressively elevating whole blood metal ion levels	22 hips (38%)
Symptomatic hip and elevated whole blood metal ion levels	14 hips (24%)
Symptomatic hip, not elevated whole blood metal ion levels	5 hips (9%)
Pseudotumor and elevated whole blood metal ion levels	14 hips (24%)
Aseptic cup loosening	3 hips (5%)
Total	58 hips (100%)

### Follow-up

After the recall of DePuy ASR hip arthroplasties and the Medicines and Healthcare products Regulatory Agency (MHRA) medical device alert regarding MoM hip arthroplasties, a systematic screening programme was launched at our institution [31, 32]. All patients with MoM hip arthroplasty were included in the programme. Patients were given Oxford Hip Score questionnaire, examined physically (including the Harris Hip Score) and whole blood chromium and cobalt ion levels were measured [33, 34]. Hip and pelvic radiographs were taken before each visit. In addition, all patients were referred for Metal Artifact Reduction Sequence MRI (MARS-MRI), unless there were contraindications, in which case patients were referred for ultrasound imaging of the hips. Findings were classified using a previously published pseudotumour classification [4]. For the purposes of the study, pseudotumours were considered as fluid-filled or solid soft-tissue masses adjacent to the articulation (classes 1, 2A, 2B or 3).

### Indications for revision surgery

Revision surgery was considered if 1) a clear pseudotumour (class 2A,2B or 3) [4] was observed on cross-sectional imaging regardless of symptoms or whole blood (WB) metal ion levels; or 2) the patient had elevated WB metal ion levels and hip symptoms despite normal findings in cross-sectional imaging; or 3) the patient had a continuously symptomatic hip or progressive symptoms regardless of imaging findings or metal ion levels; or 4) the patient had progressively increasing blood metal ion levels, even without symptoms or findings in cross-sectional imaging. Symptoms included hip pain, discomfort, sense of instability, and/or impaired function of the hip and sounds from the hip (clacking, squeaking). WB metal ion levels were regarded as being elevated if either chromium or cobalt exceeded 5 ppb [35].

### Bearing wear analysis

The volume of material loss from the cup and head bearing surfaces was measured using a Zeiss Prismo (Carl Zeiss Ltd., Rugby, UK) coordinate measuring machine (CMM). A total of 400 polar scan lines on each surface were defined and up to 30,000 data points captured using a 2 mm ruby stylus; protocols for this method have been previously published [36]. An iterative least square fitting method was used to analyze the raw data captured by the CMM and the unworn geometry of the bearing surface was used to map regions of material loss from which the total volumetric loss was calculated for each component. Total wear volume was calculated by combining head and cup wear volumes for each patient.

### Histopathological analysis of the periprosthetic tissue

During every hip revision, samples of the inflamed synovia or pseudotumor capsule were obtained. For histopathological analysis, each tissue sample was formalin fixed and embedded in paraffin. Several 10 μm microtome sections were made and stained with standard hematoxylin and eosin staining. The sections were examined histologically under transmitted light with a Nikon Eclipse 50i microscope (Nikon Corporation, Shinagawa, Tokyo, Japan). The sections were graded by a senior musculoskeletal pathologist (JP) using scoring principles adopted from the study by Natu et al. [7]. The pathologist was blinded from clinical patient characteristics.

The Natu grading consisted of following parameters: 1) macrophage sheet thickness, 2) lymphocyte cuff thickness, 3) degree of necrosis, 4) presence of plasma cells, 5) presence of diffuse lymphocytic infiltrate, 6) presence of germinal centers, and 7) presence of granulomas. Thickness of histiocyte sheets was calculated using a graticule and graded 0–3 (absent, < 1 mm, 1–2 mm, > 2 mm). Lymphocyte cuff thickness was also calculated using a graticule. An average of five measurements was taken and graded as 0–3 (absent, 0.25 mm, 0.25–0.75 mm, > 0.75 mm). The extent of overall tissue necrosis in a sample was graded based on the surface necrosis typing according to Davies et al. [37]. Type 1 surface contains intact synovial epithelium. Type 2 surface shows loss of synovial epithelial cells without fibrin deposition. In type 3 surface there is fibrin deposition and in type 4 surface there is extensive necrosis and loss of architecture. The extent of type 4 surface necrosis was used to grade the overall tissue necrosis in a given sample, as described by Natu et al. [7]. In grade 4 necrosis, more than 75% of the tissue sample showed type 4 surface necrosis. In grade 3 necrosis, between 25 and 75% showed type 4 surface necrosis. In grade 2 necrosis either less than 25% of the tissue showed type 4 surface necrosis or the tissue showed type 3 surface. In grade 1 necrosis, the sample consisted of type 2 surface.

### Statistical analysis

Statistical analyses were performed using SPPS software (IBM Corp. Released 2012. IBM SPSS Statistics for Windows, Version 21.0. Armonk, NY: IBM Corp.). Medians, ranges and interquartile ranges were calculated for total wear volume in both hips (skewed distribution). The statistical significance of the difference in wear volume between the higher and lower wearing side was tested using Wilcoxon signed ranks test (related samples). Mann-Whitney U-test was used to test the difference in wear volume distribution between the hips in patients with symmetric versus asymmetric histological and imaging findings (independent samples). The differences in histological findings between left and right hips were compared and number of patients with identical findings, patients with a difference of one point, difference of two points between the sides etc. calculated. The statistical significance of the difference in histological findings between the sides was tested with marginal homogeneity test except the difference in presence of germinal centers which did not fill the test requirements and McNemar test was used instead [38]. Whether presence of MRI-confirmed pseudotumour was similar between left and right sides was tested using McNemar test (related samples).

## Results

Thirteen of the 29 patients included in the study were females (45%). Mean age of the patients was 61.7 years (SD 8.3 years) at the time of the first revision operation and 63.1 years (SD 8.5 years) at the time of the second revision operation, respectively. On average, the first hip was revised 4.5 years (SD 1.29 years) and the second hip 5.8 years (SD 1.8 years) after the primary operation.

**Fig. 1:**
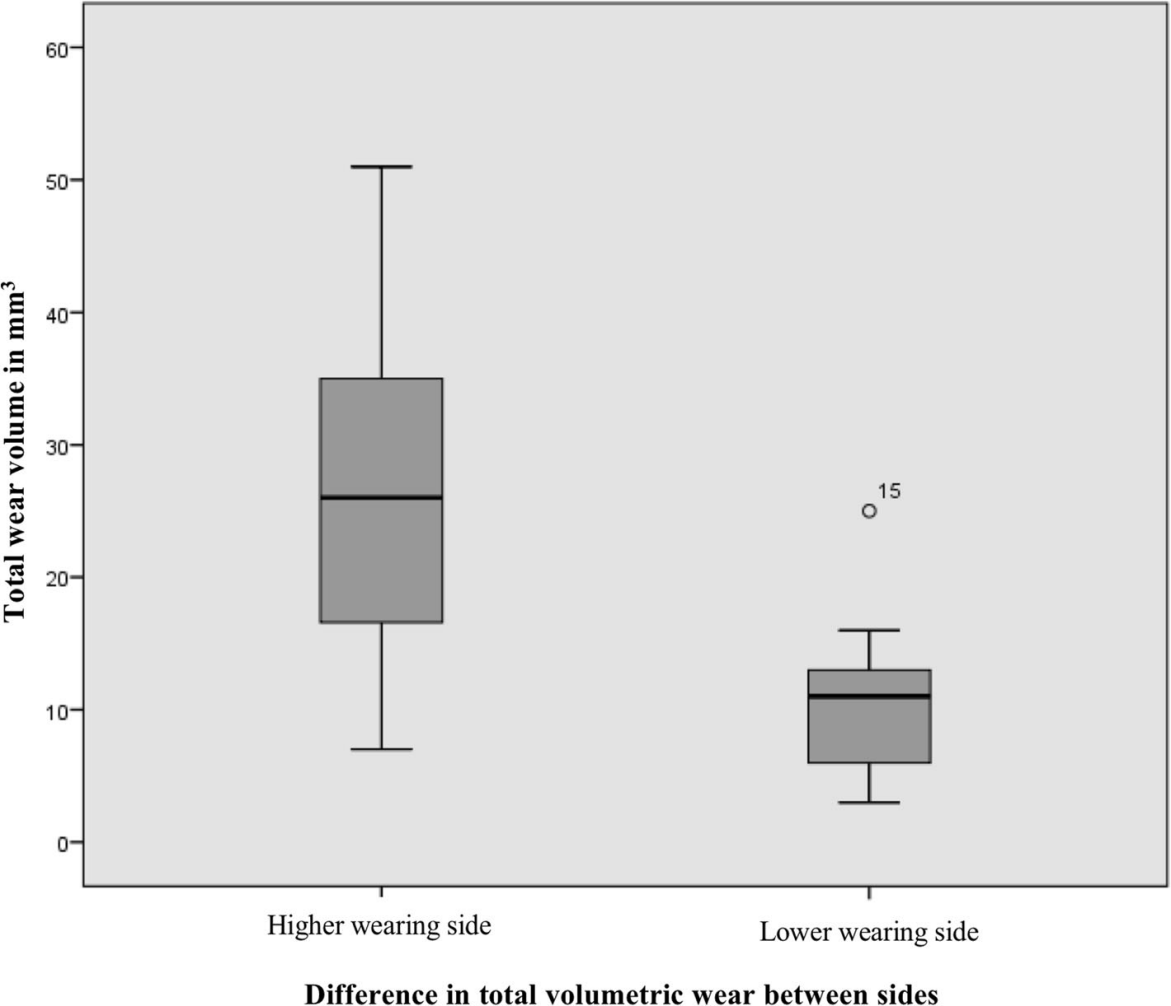
The difference in total wear volume between higher and lower wearing sides

Component wear was available bilaterally for 17 (59% of all) patients. Total wear volume in either hip ranged from 3 mm^3^ to 94 mm^3^ (median 13 mm^3^, IQR 10 to 32 mm^3^). The median difference in wear volume between higher and lower wearing side was 15.35 mm^3^ (range 1 to 39 mm^3^, IQR 6 to 23 mm^3^) (*p* < 0,001). This difference is illustrated in Fig. 1. The median ratio of total wear volume between the hips was 2.0 (range 1.09 to 10.0, IQR 1.67 to 3.72). In 9 of the 17 (53%) patients with wear data available, the ratio of wear was 2.0 or greater, ie. there was at least two-fold difference in the wear volume between the hips.

The variability of histological findings was high (Table 2). Most hips evinced mild-to moderate macrophage and lymphocyte infiltration, while in some patients there was heavy infiltration of either macrophages or lymphocytes but not both simultaneously. The degree of necrosis was approximately evenly distributed in all five grades. Majority of patients evinced no plasma cells, diffuse lymphocytic infiltration, germinal centers or granulomas.

**Table 2:** Between-subject differences in histological findings

Histological finding	Right hips	Left hips
Macrophage sheet thickness		
*0 (absent)*	1 (3.4%)	1 (3.4%)
*1 (< 1 mm)*	19 (65.5%)	24 (82.8%)
*2 (1–2 mm)*	7 (24.1%)	4 (13.8%)
*3 (> 2 mm)*	2 (6.9%)	0 (0.0%)
Lymphocyte cuff thickness		
*0 (absent)*	13 (44.8%)	13 (44.8%)
*1 (0.25 mm)*	11 (37.9%)	11 (37.9%)
*2 (0.25–0.75 mm)*	5 (17.2%)	4 (13.8%)
*3 (> 0.75 mm)*	0 (0.0%)	1 (3.4%)
Degree of necrosis		
*0*	6 (20.7%)	0 (0.0%)
*1*	4 (13.8%)	3 (10.3%)
*2*	8 (27.6%)	7 (24.1%)
*3*	7 (24.1%)	5 (17.2%)
*4*	4 (13.8%)	14 (48.3%)
Presence of plasma cells		
*No*	23 (79.3%)	22 (75.9%)
*Yes*	6 (20.7%)	7 (24.1%)
Presence of diffuse lymphocytic infiltration		
*No*	22 (75.9%)	20 (69.0%)
*Yes*	7 (24.1%)	9 (31.0%)
Presence of germinal centers		
*No*	27 (93.1%)	29 (100%)
*Yes*	2 (6.9%)	0 (0.0%)
Presence of granulomas		
*No*	23 (79.3%)	28 (96.6%)
*Yes*	6 (20.7%)	1 (3.4%)

The congruence of histological findings between the left and the right hips is presented in Table 3. In majority of the histological features and also in majority of the patients, there were no differences between the hips (*p* > 0.05 for all comparisons). These features included macrophage sheet thickness, perivascular lymphocyte cuff thickness, presence of plasma cells, presence of diffuse lymphocytic infiltration and presence of germinal centers. In lymphocyte cuff thickness the difference between the sides was at most 1 point. In macrophage sheet thickness the findings were similar in 18 patients, differed by 1 point in 9 patients and differed by 2 points in 2 patients, respectively. The only histological findings that statistically significantly differed between the hips were grade of necrosis (*p* < 0.01) and presence of granulomas (*p* = 0.025). In the grade of necrosis there was a wide distribution in the difference between the sides. In those patients with granuloma present on one side only, the granuloma was always on the higher-wearing side. When comparing all hips, those hips with a granuloma (*n* = 5) had a median total wear volume of 35 mm^3^ (range 15.0 to 111.0) and those hips with no granuloma (*n* = 39) had a median total wear volume of 15 mm^3^ (range 3.0 to 94.0) (*p* = 0.059 for comparison). In the grade of necrosis, the higher grade was not always on the higher wearing side. In any of the histological findings, the symmetry or asymmetry of findings between left and right sides was not associated with a difference in the distribution of wear volume between the sides (Table 4). All patients had at least two histological variables with similar findings on both hips. Majority of the patients (75.9%) had four or more histological variables with similar findings on both sides (Table 5). There were no differences in the similarity or dissimilarity of histological findings between left and right hips in males versus females (Table 6).

**Table 3:** Congruence in histological grading between left and right hips (within-subject)

	Difference in histological grading between left and right sides					
	No difference	1 p	2p	3p	4p	Scale
Macrophage sheet thickness	18 (62%)	9 (31%)	2 (7%)	–		0–3 p
Lymphocyte cuff thickness	14 (48%)	15 (52%)	–	–		0–3 p
Degree of necrosis*	6 (21%)	10 (34%)	9 (31%)	1 (3%)	3 (10%)	0–4 p
Presence of plasma cells	26 (90%)	3 (10%)				Yes/no
Presence of diffuse lymphocytic infiltration	19 (66%)	10 (34%)				Yes/no
Presence of germinal centers	27 (93%)	2 (7%)				Yes/no
Presence of granulomas*	24 (83%)	5 (17%)				Yes/no

Percentages represent proportion of all patients. In variables marked with * there was a statistically significant (*p* < 0.05) difference between the sides (see results)

**Table 4:** Median differences in total wear volumes between the sides (mm^3^)

Histology between sides	Symmetrical	Asymmetrical	*P*-value
Macrophages	9.0	18.7	0.40
Lymphocytes	18.0	12.7	0.89
Necrosis	32.0	12.7	0.35
Plasma cells	16.0	5.3	0.24
Diffuse lymphocytes	16.0	6.3	0.48
Germinal centers	15.7	23.0	0.71
Granulomas	15.35	16.1	0.70

Median differences in total wear volumes between the sides in patients with symmetrical histological findings versus patients with asymmetrical histological findings. Only patients with complete wear data are included (*n* = 17)

**Table 5:** The degree of similarity between the hips measured by the number of histological variables with similar findings on both sides in each patient

Histological variables with symmetric findings on both sides	Number of patients	Percentage of patients
0	0	0%
1	0	0%
2	2	6.9%
3	5	17.2%
4	6	20.7%
5	6	20.7%
6	9	31.0%
7	1	3.4%
	Total 29	Total 100%

**Table 6:** Comparison of similar versus not similar histological findings between the sides in males and females

Histological variable	Symmetric findings on both hips	Males	Females	*P*-value for the difference between males and females
Macrophage sheet thickness	Yes	11 (69%)	7 (54%)	0.46
	No	5 (31%)	6 (46%)	
Lymphocytic cuff thickness	Yes	9 (56%)	5 (38%)	0.46
	No	7 (44%)	8 (62%)	
Degree of necrosis	Yes	4 (25%)	2 (15%)	0.66
	No	12 (75%)	11 (85%)	
Presence of plasma cells	Yes	14 (88%)	12 (92%)	0.58
	No	2 (12%)	1 (8%)	
Presence of diffuse lymph.	Yes	12 (75%)	7 (54%)	0.27
	No	4 (25%)	6 (46%)	
Presence of germinal centers	Yes	15 (94%)	12 (92%)	1.0
	No	1 (6%)	1 (8%)	
Presence of granulomas	Yes	15 (94%)	9 (69%)	0.14
	No	1 (6%)	4 (31%)	

Bilateral MRI classification for the presence of pseudotumours was available for 25 patients (86% of all patients). 18 patients (72% of the classified) had either bilateral pseudotumours or no pseudotumours at all on either side, ie. the hips were symmetrical in regard to pseudotumour. There was no statistically significant difference in the presence of pseudotumour between the sides (*p* = 0.13). Of those 18 patients, 7 had pseudotumour on both sides (of which two were identical by exact classification) and 11 had no pseudotumour on either side. Patients with asymmetrical pseudotumour finding between the sides evinced similar distribution of total wear volume between the sides as those patients with symmetrical pseudotumour findings (Table 7). In addition, there were no differences in the total wear volumes of the hips in patients with pseudotumour on both sides (median 20.0 mm^3^, range 9.0 to 111.0) versus no pseudotumour on either side (median 16.30 mm^3^, range 3.0 to 51.0) (*p* = 0.28 for comparison).

**Table 7:** Pseudotumour finding and wear volumes between the sides

Pseudotumour	Symmetrical	Asymmetrical	*P*-value
Median difference in total wear volume between the sides (mm^3^)	12.7	13.5	0.79

The distribution of wear volume between left and right sides is similar in patients with symmetrical and asymmetrical pseudotumour findings between the sides. Only patients with complete wear data are included (*n* = 17)

## Discussion

In the present study, we found that there were notable differences in the histological findings between patients revised for ARMD, ie. the between-subject variability was high. Heterogeneity has been characteristic for the results of ARMD research [16]. Most importantly, however, we found no statistically or clinically significant differences in most of the histological and imaging findings between left and right hips of the same patient, meaning that the within-subject variability in histological and imaging findings was low. Further, majority of the patients had similar findings on both hips in several key histological variables. This was despite the fact that there was a clinically and statistically significant difference in the amount of wear volume between the sides, ie. there was a difference in the extrinsic factor between the sides. There are no clearly defined boundaries for abnormal versus normal wear, but volumetric wear rates exceeding 1 mm^3^/year are generally considered abnormal [39]. As the median difference of 15.4 mm^3^ in wear volume between the sides measured in our study translates into remarkably abnormal yearly volumetric wear rate needed to generate that difference, we thus feel safe to consider the difference in median wear volume between the sides clinically significant.

The contribution of host-specific factors in the pathogenesis of ARMD has been suggested in numerous previous studies, likely observed as patient susceptibility of different levels [8, 13, 14, 24–28]. However, to the best of our knowledge there are no previous studies that would have actually assessed the role of intrinsic factors in the pathogenesis. On the contrary, there are many studies that have investigated implant wear and the development of ARMD, however, results of these studies are very discrepant. High wear or high blood metal ion levels resulting from high wear are associated with the development of ARMD [10, 40]. However, adverse reactions have been noted in patients with both high and low wearing hip implants [8, 11, 13, 25, 41]. In a systematic review by Campbell et al. no clear dose-response relationship between wear and ARMD could be established [16]. We observed symmetry of histological findings between left and right hips despite differing amounts of wear. In addition, the distribution of wear volume between the sides was similar in patients with symmetrical versus asymmetrical histological and imaging findings. Further, patients with bilateral pseudotumours had similar amounts of wear volumes in their hips as did patients with no pseudotumour on either side. Our finding suggests that there are intrinsic factors that markedly contribute to the pathogenesis of ARMD, dictating the type of tissue response and development of pseudotumours, in addition to extrinsic factors such as volume of the metal wear debris. Further, it is likely that there are differences in these intrinsic factors between patients as some develop aggressive tissue responses despite low-wearing implant while some tolerate large amounts of wear. Various terms have been used to describe this phenomenon, for example patient susceptibility [13]. Clinicians should bear in mind that some patients with low wearing implants (low blood metal ion levels) can still be at risk for ARMD due to higher than average patient susceptibility.

A cohort of patients with bilateral MoM hips forms an excellent research frame to investigate and compare the role of intrinsic and extrinsic factors in the pathogenesis. We are aware of only three previous studies that compare characteristics of ARMD between the sides in patients with bilateral MoM hip replacements. Madanat et al. compared MRI findings between left and right hips in patients with bilateral MoM hip replacements [42]. They found that the soft tissue reaction observed in MRI was symmetrical between the sides in most patients, both in sequentially and simultaneously implanted hips. In support of their findings, we report similar symmetry for the presence of MRI-confirmed pseudotumour between the sides. Another study by Pandit et al. consisted of four revised patients with bilateral MoM hips [43]. All patients had developed a necrotic pseudotumor in both hips. In histopathological analysis, both hips of each patient had similar findings (necrosis, macrophages, lymphocytes). However, no wear data was included in the study and the histology was descriptive, not semiquantitatively scored. A recent study by Uchihara et al. included patients with both uni- and bilateral MoM hips that had been revised for ARMD [44]. They compared histological findings between left and right hips in the bilateral patients as well as histological findings between unilateral and bilateral patients. In addition, time-to-failure was compared between these two groups. The histological findings (necrosis, macrophages, lymphocytes) between left and right hips of the bilateral patients were found to be symmetrical in majority of the cases, similar to the findings of the present study. However, we observed that there were differences in the grade of necrosis between the sides while Uchihara et al. did not semiquantitatively grade the necrosis. Further, there were no differences in the histological findings or time-to-failure between uni- and bilateral patients in their study. Uchihara et al. concluded that the implantation of a MoM hip does not appear to lead to sensitization to metal debris that would in turn lead to poor clinical performance or different tissue response in the second MoM hip. However, they did not discuss the significance of their findings in the context of intrinsic factors contributing to the similarity of the tissue response between the hips in bilateral patients. Further, their sample size was rather small (10 patients) and no wear data of the MoM hips was presented in the study. These three previous studies conducted on bilateral MoM patients are in agreement with our findings and support the hypothesis of an individual host response dictated by intrinsic factors as a significant contributor in the development of soft tissue reactions leading to failure of the hip.

The pathogenesis of ARMD is poorly understood, but at least three different mechanisms of failure have been suggested: 1) type IV hypersensitivity response to metal wear debris with adaptive immunity involvement, 2) foreign-body response to metal wear particles reflecting innate immunity and 3) direct cytotoxic effect of metal ions [6, 8, 45]. To what degree the tissue response depends on the amount of wear and to what degree on the host-specific intrinsic factors is not well understood. We hypothesized that components of the innate response (macrophages, granulomas, necrosis) are more closely related to extrinsic factors and components of the adaptive response (lymphocytes, germinal centers and plasma cells) to intrinsic factors such as genetic predisposition to metal hypersensitivity. We found that the grade of tissue necrosis and presence of granulomas differed between the sides in most patients. Granulomas were always present on the higher wearing side. Further, when analyzing all hips as a group, we found that there was a trend for higher total wear volume in hips with a granuloma compared to those hips with no granuloma. However, this difference did not quite reach statistical significance. Granulomas are considered to form as a response to high numbers of metal particles in tissues [46]. Our results support this idea. Still, in the present study granulomas were not present in the majority of the hips. We suggest that there is a certain threshold for tissue metal content needed for granulomas to develop as a response. Whether this threshold is dependent on intrinsic factors, particle size, non-particulate metal debris or particle type is not understood and requires further research. The metal ions released from implants are known to cause dose-dependent cytotoxicity in-vitro [47]. Also, we and others have previously shown that implant wear correlates with necrosis of the periprosthetic tissues [6, 48]. Thus, it seems likely that extrinsic factors, mainly implant wear, are more important in the development of tissue necrosis and granulomas than intrinsic factors, ie. patient susceptibility. However, opposite to our hypothesis, the grade of macrophage sheet thickness did not differ between the sides. This would suggest that the macrophage response (innate) is mostly determined by host-specific factors instead of extrinsic factors such as volume of the wear debris. However, there are limitations in our methodology. We did not directly measure the number of macrophages, instead, we measured the thickness of the macrophage sheets. It is possible that the infiltration penetrates deep in the tissue but is not dense. We observed that there were no statistically significant differences in the amounts of lymphocytes and presence of plasma cells and germinal centers between the hips, despite markedly different wear volumes in most of these patients. These parameters belong to the adaptive immune system which is considered host-specific. Thus, it makes sense that they are expressed symmetrically. In some studies, it has been found that low wear is associated to adaptive lymphocytic response and high wear to innate, macrophage dominated foreign-body response [6, 8, 21]. These associations have been weak, however. In addition, disagreeing findings have been published [19, 22]. We suggest that host-specificity of the intrinsic factors leads to differences in the tissue response between individuals no matter what the wear. This likely contributes to the poor association between the amount of wear and type of inflammatory tissue response in previous literature.

Our study is not without limitations. First, the sample size in our study is rather small. However, it is clearly the largest in any published study dealing with this issue so far. Second, not all hips were analyzed for bearing wear volume. However, it must be noted that large patient cohorts with clinical information, laboratory and imaging findings, tissue samples and also retrieval analyses available, are not easily available anywhere globally. Further, our patient cohort is free of selection bias as all patients have been primarily operated and followed-up thereafter at our institution with no referrals from other centers. Thirdly, we were not able to analyze the volume of the material loss from the trunnion in those patients with ASR XL hip implants. However, the volume of the material loss from the trunnion is known to be less than that from the bearing couple [49]. Fourth, we used surrogate markers (semiquantitative histology) to indirectly investigate the presence of intrinsic factors contributing to the response. Measuring variability in signaling pathways provide more direct evidence, but was out of the scope of the current study. Besides, histological methods are well-documented and there is vast amount of literature regarding ARMD histology. However, it is not yet well understood which signaling pathways are important in the development of ARMD and thus a comprehensive study of such would not be realistic. Our study offers novel insight into the role of intrinsic versus extrinsic factors in the pathogenesis of ARMD and is the largest bilateral patient cohort published on the subject. Further, our study is the first one to include wear data.

## Conclusion

In conclusion, intrinsic host-specific factors most likely contribute to the development of ARMD in addition to extrinsic factors such as implant wear debris. Further, it is likely that there are differences in these host-specific factors between patients, manifesting as susceptibility to metal debris of variable degree. Clinicians should bear in mind that patients may have different responses to the same amount of wear debris, usually measured as blood metal ion levels. Some patients may tolerate high amounts of metal debris and some patients may develop even severe adverse tissue responses in the presence of a low-wearing hip implant. Also, bilateral MoM patients with failure on one side will likely develop a similar tissue response on the other side as well. This should be accounted for in the follow-up of patients with bilateral MoM hip replacements. In future studies, it is important to search for possible biomarkers that would predict the severity and type of the intrinsic response, in other words, patient susceptibility. Further, it is important to understand the true nature of ARMD in order to be able to design safer bearing couples in the future.

## Additional files

Additional file 1: Between-patient and within-patient variability explained in detail. (DOCX 65 kb)

Additional file 2: Flow chart of the patient selection for the study. (TIF 57 kb)

## Abbreviations

ARMD: Adverse Reaction to Metal Debris
ASR: Articular Surface Replacement
CMM: Coordinate Measuring Machine
MARS-MRI: Metal Artifact Reduction Sequence MRI
MHRA: Medicines and Healthcare products Regulatory Agency
MoM: Metal-on-Metal
