# Supplementary material for: Host-specific factors affect the pathogenesis of adverse reaction to metal debris
Source: BMC Musculoskelet Disord. 2019 May 4;20:195. doi: 10.1186/s12891-019-2578-0 (PMC6499989; doi:10.1186/s12891-019-2578-0)
Supplement: Supplementary file 1 — Between-patient and within-patient variability explained in detail. (DOCX 65 kb) [file 12891_2019_2578_MOESM1_ESM.docx]

**
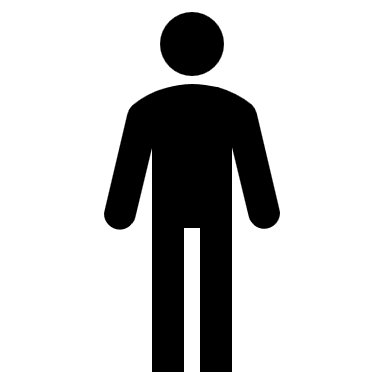

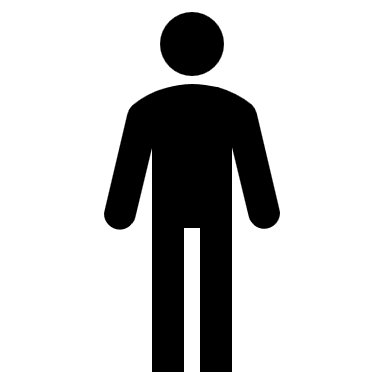
**

Right hip Right hip

The amount of wear volume = **external factor**

Tissue response

Tissue response

**Between-patient variation (in this case high)**

**
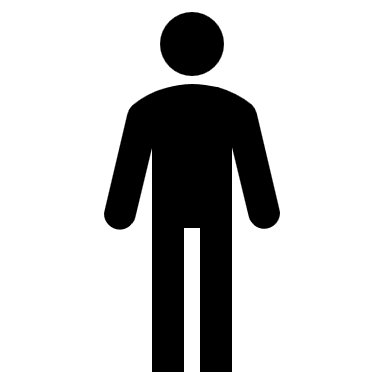
**

Right hip Left hip

The amount of wear volume = **external factor**

**Intrinsic factors**

Tissue response

Tissue response

**Within-patient variation (in this case low)**

**Investigating individual patients, ie. the cases are independent**

Assumption 1: Each patient has one hip contributing to the study. We measure continuous outcome parameter X (tissue response).

Remark 1: When each patient contributes one hip, all the variation observed in X is due to the *between-patient* *variation*(left picture).

Assumption 2: The external factor Z (the amount of wear volume) is known to have an effect. The Z is same for all patients. If observed variation is small, there is no intrinsic factor having effect on X, ie. the X is influenced only by Z. If variation is, however, high among patients, the intrinsic factor has an effect on X.

Conclusion 1: If between-patient variation is small while Z remains same, there is no intrinsic factor at play.

Remark 2: Usually no external factors are essentially same for all patients but they change from patient to patient in a cohort study. Problem: we cannot ascertain for intrinsic factors.

**Comparing two hips from the same patients (dependent cases)**

When we compare hips from same patient we have eliminated the between-patient variation.

Remark 1: If *within-patient variation, i*e. discrepancy between left and right hip in a same patient*,* is low regardless of the different levels of the external factor Z between the hips on a same patient, the intrinsic factor has greater effect on X than does Z. The X is mainly determined by the intrinsic factor. (Right picture)
