# Supplementary figures and images for: Host-specific factors affect the pathogenesis of adverse reaction to metal debris
Source: BMC Musculoskelet Disord. 2019 May 4;20:195. doi: 10.1186/s12891-019-2578-0 (PMC6499989; doi:10.1186/s12891-019-2578-0)

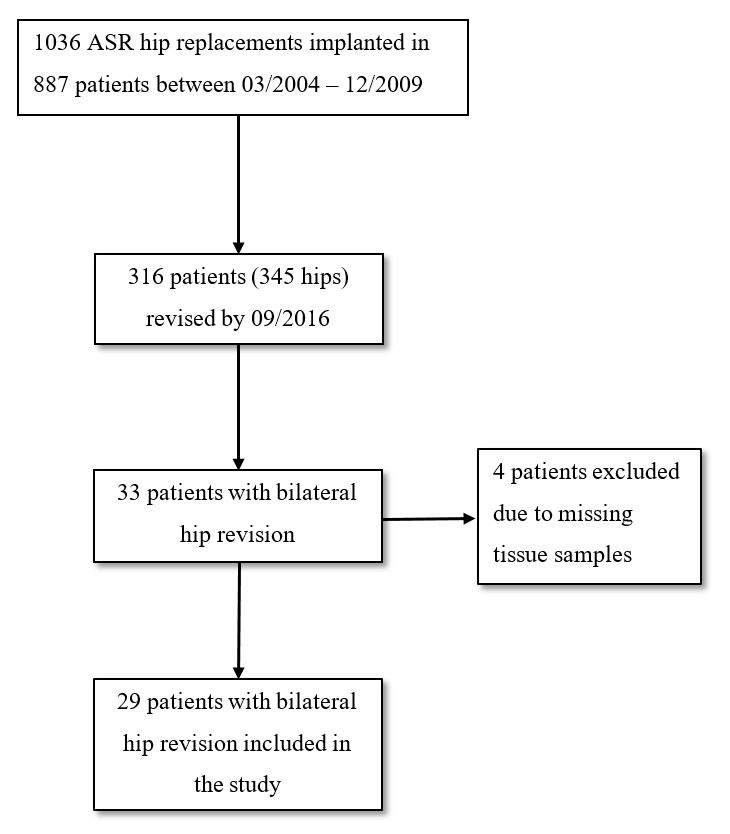

Supplement: Supplementary file 2 — Flow chart of the patient selection for the study. (TIF 57 kb) [file 12891_2019_2578_MOESM2_ESM.tif]
